# Effects of Dietary Fibre (Pectin) and/or Increased Protein (Casein or Pea) on Satiety, Body Weight, Adiposity and Caecal Fermentation in High Fat Diet-Induced Obese Rats

**DOI:** 10.1371/journal.pone.0155871

**Published:** 2016-05-25

**Authors:** Clare L. Adam, Silvia W. Gratz, Diana I. Peinado, Lynn M. Thomson, Karen E. Garden, Patricia A. Williams, Anthony J. Richardson, Alexander W. Ross

**Affiliations:** 1 Obesity & Metabolic Health Division, Rowett Institute of Nutrition & Health, University of Aberdeen, Aberdeen, Scotland, United Kingdom; 2 Gut Health Division, Rowett Institute of Nutrition & Health, University of Aberdeen, Aberdeen, Aberdeen, Scotland, United Kingdom; Western University of Health Sciences, UNITED STATES

## Abstract

Dietary constituents that suppress appetite, such as dietary fibre and protein, may aid weight loss in obesity. The soluble fermentable dietary fibre pectin promotes satiety and decreases adiposity in diet-induced obese rats but effects of increased protein are unknown. Adult diet-induced obese rats reared on high fat diet (45% energy from fat) were given experimental diets *ad libitum* for 4 weeks (*n* = 8/group): high fat control, high fat with high protein (40% energy) as casein or pea protein, or these diets with added 10% w/w pectin. Dietary pectin, but not high protein, decreased food intake by 23% and induced 23% body fat loss, leading to 12% lower final body weight and 44% lower total body fat mass than controls. Plasma concentrations of satiety hormones PYY and total GLP-1 were increased by dietary pectin (168% and 151%, respectively) but not by high protein. Plasma leptin was decreased by 62% on pectin diets and 38% on high pea (but not casein) protein, while plasma insulin was decreased by 44% on pectin, 38% on high pea and 18% on high casein protein diets. Caecal weight and short-chain fatty acid concentrations in the caecum were increased in pectin-fed and high pea protein groups: caecal succinate was increased by pectin (900%), acetate and propionate by pectin (123% and 118%, respectively) and pea protein (147% and 144%, respectively), and butyrate only by pea protein (309%). Caecal branched-chain fatty acid concentrations were decreased by pectin (down 78%) but increased by pea protein (164%). Therefore, the soluble fermentable fibre pectin appeared more effective than high protein for increasing satiety and decreasing caloric intake and adiposity while on high fat diet, and produced a fermentation environment more likely to promote hindgut health. Altogether these data indicate that high fibre may be better than high protein for weight (fat) loss in obesity.

## Introduction

Dietary constituents that are able to enhance satiety and promote weight loss provide an attractive proposition for obesity management. The two macronutrients most commonly associated with increased satiety are dietary fibre and protein, yet there is a lack of publications comparing and defining their efficacy in obese subjects. These biological responses are most usefully and accurately determined in the controlled circumstances of laboratory animal models before advising human dietary intervention trials. Thus, we have recently demonstrated how addition of the soluble fermentable dietary fibre pectin to a high fat diet increases satiety, decreases caloric intake and leads to weight (body fat) loss in diet-induced obese (DIO) rats [1]. In a similar experimental paradigm, we now investigate the individual and interactive effects of supplementary pectin and increased protein of either animal (casein) or plant (pea) origin.

There are multiple health benefits for humans with obesity from the dietary incorporation of fibre supplements, including increased satiety and weight loss, but these have not been clearly quantified in the literature [2]. Nonetheless there is good evidence for increased dietary fibre of various types protecting against the development of hyperphagia and obesity in rats and mice fed high fat diets [3–6] and our earlier study demonstrated the efficacy of supplementation with the dietary fibre pectin in promoting satiety, hypophagia and weight (fat) loss in rats that were already obese at the start of dietary intervention [1]. The increased intake of dietary fibre in these rodent models is associated with increased secretion of gut satiety hormones, notably PYY and GLP-1 [1, 3, 7, 8].

High protein diets (i.e. with protein providing 30–40% food energy) have also emerged over the last decade as a means to achieve weight loss, with increased satiety being the key underlying mechanism [9–11]. Increased intake of dietary protein is associated with increased release of the gut satiety hormone PYY in humans and mice, while exogenous PYY reverses the hyperphagic obesity seen in PYY-knockout mice [9]. Furthermore, DIO rats given high protein diet (52% energy from protein) for up to 4 weeks showed decreased body weight and caloric intake and increased circulating PYY concentrations [12].

However, a recent meta-analysis found persistent benefits of high protein for weight loss in humans only in highly controlled feeding studies, with a lack of dietary compliance shown by free-living adults [13]. Moreover, long-term high protein intake is detrimental to renal health, as demonstrated in pigs and rats given diet with 35% energy from protein [14, 15], and is potentially harmful to colonic health, as shown in rats [16] and humans [17]. The harmful colonic effects are largely attributable to changes in the fermentation pattern and metabolites of the gut microbiota when undigested protein reaches the large intestine. Conversely, increased dietary fibre intake promotes a healthy colonic environment, with its cancer-protective effects linked to favourable products of fermentation [18]. Consequently, it has been suggested that inclusion of sufficient fibre or digestion-resistant carbohydrate in high protein weight-loss diets could counteract some of the adverse consequences on gut health [17]. Whereas investigating fermentation products in humans relies on faecal analyses, in the present animal model we are able to measure concentrations of the main fermentation products directly within the large intestine where they are produced.

The products of fermentation, the short-chain fatty acids (SCFAs), are not only critical for colonic health and suppression of inflammation and cancer [19] but they also play a significant role in appetite regulation and energy homeostasis [20]. Amongst other beneficial effects on energy balance, SCFAs have been shown to stimulate the secretion of satiety hormones PYY and GLP-1, and acetate reaching the peripheral circulation can stimulate anorectic hypothalamic pathways in the brain [20]. The implications of altering SCFA production through different combinations of elevated dietary fibre and protein are likely to be important. Furthermore, the type as well as the quantity of protein is likely to influence the fermentation processes. For example, proteins with different digestibility patterns will affect fibre fermentation, and thereby the concentrations of the different fermentation products, by providing the hindgut microbes with different amounts of nitrogen [21, 22]. However, there is no consensus on differing satiating powers of different protein sources. In short-term human studies, there was no difference between beef vs soy protein in one study [23] but casein and pea were both more satiating than whey in another [24], whereas food intake is decreased in rats when increased protein is provided as whey, but not as soya [25]. To examine this further, it was therefore pertinent to include in our animal model two very different proteins, of animal vs plant origin, at the same % energy dietary inclusion rate.

In summary, this experiment compared the separate and combined effects of increased dietary soluble fibre (pectin) and animal (casein) or plant (pea) protein in young adult high fat diet-induced obese rats in terms of food intake, satiety, weight loss, body composition, circulating satiety and metabolic hormones and hindgut fermentation products.

## Materials and Methods

### Ethics statement

All animal experimental procedures met institutional and national guidelines for the care and use of animals. They were licensed by the UK Home Office Animals (Scientific Procedures) Act, Amended 2012, under Project License 60/4282 and were approved by the local ethical review committee at the University of Aberdeen Rowett Institute of Nutrition & Health (approval number 301013CA). Rats were euthanised by decapitation under general inhalation anaesthesia (isoflurane; IsoFlo, Abbott Animal Health, Maidenhead, Berkshire, UK).

### Diets

Diets were pelleted, *ad libitum*-fed, based on purified AIN-93 (American Society for Nutrition, Bethesda, MD, USA) and made and supplied by Special Diet Services Ltd, Witham, Essex, UK. The high fat diet given to all rats in the rearing period and for one group during the main experiment was a standard purified diet with 45% energy from fat (HF). This diet contains 5–6% w/w insoluble dietary fibre cellulose and provides 20% energy from protein. In experimental high fibre diets used herein the cellulose was replaced by 10% w/w soluble fibre pectin (P; Solgar Apple Pectin, Revital Ltd., Ruislip, Middlesex UK) and in experimental high protein diets the dietary protein content was doubled to provide 40% food energy (either as standard purified casein protein or as >80% purity Chinese raw pea protein isolate, both supplied by Special Diet Services Ltd). The following 6 purified experimental diets were used: high fat diet alone (HF), high fat with high casein protein (HFHC), high fat with high pea protein (HFHP), and these diets supplemented with pectin (10% w/w pectin; HF+P, HFHC+P and HFHP+P, respectively; Table 1).

**Table 1:** Experimental diets^1^.

	Energy content (kJ/g)	% energy^2^			% w/w	Proximate analysis (%)^4^			
		Fat	Protein	CHO	Pectin^3^	Dry Matter	Nitrogen	Fat	CHO
HF	19.0	45	20	35	0	93.7	3.7	21.1	32.3
HF+P	19.5	45	20	35	10	94.4	3.8	21.9	29.6
HFHC	18.9	45	40	15	0	95.2	7.2	21.0	9.7
HFHC+P	19.6	45	40	15	10	94.4	7.6	21.3	4.4
HFHP	19.0	45	40	15	0	95.2	7.2	21.6	5.9
HFHP+P	19.7	45	40	15	10	94.5	7.6	21.1	5.9

^1^Diets were based on AIN-93M, manufactured and supplied by Special Diet Services Ltd, Witham, Essex.

^2^Atwater Fuel Equivalents.

^3^Highly esterified apple pectin (Solgar Apple Pectin; Revital Ltd., Ruislip, Middlesex UK).

^4^Standard chemical analysis of diets.

### Animals and experimental procedure

Forty-eight outbred male Sprague Dawley rats (Charles River Laboratories, Tranent, East Lothian, UK) were reared from weaning to 12 weeks of age on HF diet in order to generate DIO. Then, after 1 week’s acclimatisation to individual housing in plastic cages, they were given the experimental diets *ad libitum* for 28 days (*n* = 8/diet group). Experimental diets were introduced by mixing 50:50 with HF on day 1 and increasing to 100% over 3 days. Water was available *ad libitum*, the lighting regime was a standard 12 h light and 12 h dark, temperature was constant at 21±2°C and the relative humidity was held at 55±10%; cages contained corn cob bedding, with shredded paper for nesting and plastic tunnels for further environmental enrichment. Voluntary food intake was measured daily by weighing uneaten food each morning and body weight was measured twice a week. Body composition was measured in conscious rats at the start (day 0) and end (28 days) of the experiment by magnetic resonance imaging (MRI; EchoMRI 2004, Echo Medical Systems, Houston, TX, USA), which provided total body fat and lean mass data.

After the final MRI scan, rats were euthanised 1–3 h after lights-on; they were not fasted but were killed in their natural state during the light period when food is not consumed [26]. Final (trunk) blood samples were collected into chilled tubes containing EDTA as anti-coagulant and a peptidase inhibitor cocktail containing general protease inhibitor (cØmplete; Roche Diagnostics Ltd, Burgess Hill, West Sussex, UK) and dipeptidyl peptidase-4 inhibitor (Ile-Pro-Ile; Sigma-Aldrich, Gillingham, Dorset, UK), centrifuged immediately at 3000g for 12 min, then plasma was stored at -20°C until analysis. The gut was dissected out, wet weights were recorded immediately for stomach, small intestine, caecum and colon, and the lengths of small intestine, caecum and colon were measured.

### Plasma analyses

Hormone concentrations in plasma samples were analysed by commercial RIA kits according to the manufacturer’s instructions (Merck Millipore, Billerica, MA, USA). Total GLP-1 was measured by kit GLP1T-36HK which detects all forms of GLP-1 (lower detection limit 3 pM). Active GLP-1 was not measured because it has a very short half-life in plasma, but measurement of total GLP-1 provides an accurate indication of overall GLP-1 secretion since it includes both the intact hormone and its primary metabolite [27]. PYY was measured by kit RMPYY-68HK (lower detection limit 15.6 pg/ml), which detects both of the circulating biologically active forms of PYY, namely PYY(1–36) and PYY(3–36). Leptin was measured by kit RL-83K (lower detection limit 0.6 ng/ml) and insulin by kit RI-13K (lower detection limit 0.08 ng/ml). Plasma glucose concentrations were determined by automated KONE analyser (hexokinase method; lower detection limit 0.3 mmol/l).

### Caecal SCFA analysis

The concentrations of SCFAs produced by bacterial fermentation in caecum contents were determined by capillary gas chromatography using the method developed by Richardson et al [28]. Briefly, samples were first diluted with distilled water (1/4) and 2-ethylbutyric acid (5 mmol/L) was added as internal standard. Samples were then extracted in diethyl ether, derivatised with N-tert-butyldimethylsilyl-N-methyltrifluoroacetamide and analysed on Agilent GC HP-1 capillary columns to detect succinate, acetate, propionate, butyrate, and the branched-chain fatty acids (BCFAs) iso-butyrate, valerate and iso-valerate.

### Statistical analysis

Effects of diet on serial body weight and food intake data were analysed by repeated measures ANOVA (General Linear Model (GLM) with time, diet and their interaction as factors; Minitab Version 17, Minitab Inc., State College, PA) and the effects on all other measurements by two-way ANOVA (GLM with pectin, protein and their interaction as factors; Minitab 17) followed by Fisher pairwise comparisons. Pearson’s correlation was used to explore relationships between variables where indicated. *P* < 0.05 was taken as significant.

## Results

### Body weight, body composition and food intake

**Fig 1:**
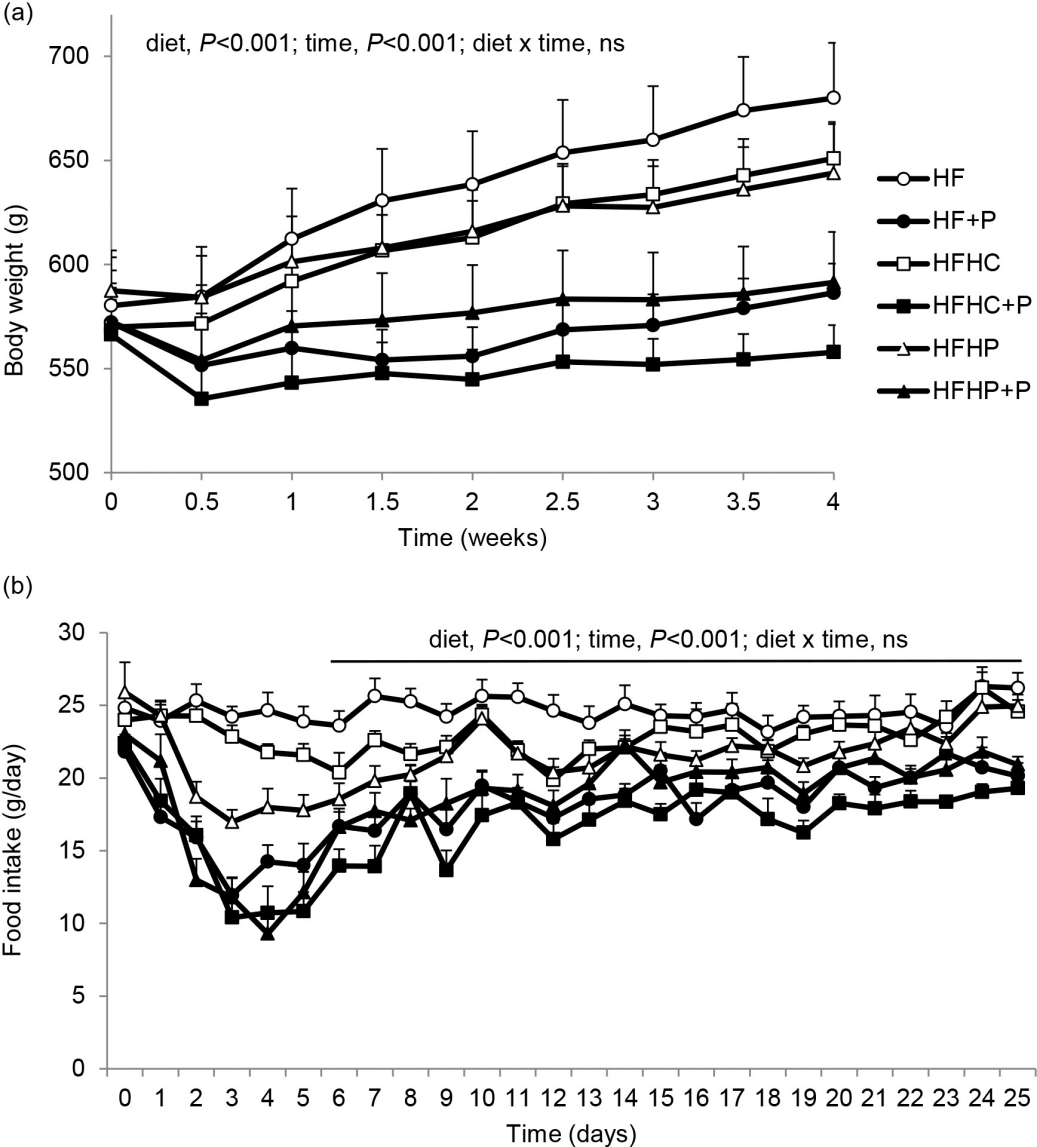
Body weight and food intake. (a) Body weights and (b) daily food intakes of diet induced obese rats fed high fat diet (HF), high fat diet with pectin (HF+P), HF with high casein protein (HFHC), HFHC with pectin (HFHC+P), HF with high pea protein (HFHP), HFHP with pectin (HFHP+P) for 4 weeks (*n* = 8 per group). Statistical analysis by repeated measures ANOVA, applied from day 6 onwards after diet acclimatisation for intake data in (b).

During the 4-week dietary intervention, body weight gain was lower in rats on the three pectin-containing (+P) diets compared with rats on HF control diet or HF with high casein (HFHC) or high pea protein alone (HFHP) (*P* < 0.001; Fig 1A).

Overall body weight gain and fat mass gain were decreased by supplementary pectin (both *P* < 0.001) and by high protein (both *P* < 0.01), with significant pectin x protein interaction (both *P* < 0.05) since the effects of pectin were greater with HFHC than HFHP diet (Table 2). Final body weight, total body fat mass and body fat percentage were significantly decreased, and lean percentage increased, by supplementary pectin (all *P* < 0.001) but not by high protein, with no pectin x protein interaction, while final total lean mass was not affected by either pectin or high protein. Pairwise comparisons between the individual diets showed that body weight gain was highest in the control HF group, no different in HFHC, but significantly lower in the three +P groups (*P* < 0.001); the intermediate value for the HFHP group was lower than HF (*P* < 0.05) but higher than the +P groups (*P* < 0.001–0.05; Table 2). There were no differences between the groups for lean mass gain, but fat mass increased substantially in HF and HFHC groups, did not change significantly in HFHP, and decreased significantly in the three +P groups (*P* < 0.001; Table 2). Thus final total body fat mass and total body fat percentage were highest in HF and HFHC, intermediate in HFHP and lowest in the three +P groups, whereas final total lean mass was not different between the groups but lean tissue percentage was lowest in HF and HFHC, intermediate in HFHP and highest in the three +P groups (Table 2).

**Table 2:** Body weight, body composition and food intake^1^.

	Diet						Significance of effects		
	HF	HF+P	HFHC	HFHC+P	HFHP	HFHP+P	Pectin	Protein	Pectin x protein
Body weight (g) -Before	574.0±22.7	552.5±18.3	565.1±18.5	562.1±17.9	580.4±20.1	567.7±26.2	n.s.	n.s.	n.s.
-After	680.0±26.5^a^	586.4±14.0^bc^	650.9±17.5^a^	557.9±13.0^c^	643.9±23.6^ab^	591.4±24.3^bc^	*P* < 0.001	n.s.	n.s.
-Change	106.0±7.9^a^	33.9±14.0^c^	85.8±5.2 ^ab^	-4.2±7.1^d^	63.5±8.3^b^	23.7±10.8^c^	*P* < 0.001	*P* < 0.01	*P* < 0.05
Total fat mass (g) -Before	95.6±9.3	92.2±13.9	103.2±12.1	90.8±10.5	106.1±14.7	90.6±9.6	n.s.	n.s.	n.s.
-After	134.1±12.5^a^	77.3±7.8^bc^	132.9±12.3^a^	65.4±7.1^c^	107.7±15.1^ab^	67.0±9.6^c^	*P* < 0.001	n.s.	n.s.
-Change	38.5±6.5^a^	-14.9±9.1^bc^	29.8±3.9^a^	-25.4±5.9^c^	1.63±5.6^b^	-23.6±6.8^c^	*P* < 0.001	*P* < 0.01	*P* < 0.05
Final total fat (%)	19.6±1.7^a^	13.1±1.2^bc^	20.2±1.5^a^	11.6±1.1^c^	16.4±1.7^ab^	11.1±1.3^c^	*P* < 0.001	n.s.	n.s.
Total lean mass (g)-Before	445.3±13.7	407.9±11.0	409.0±10.4	418.6±5.8	441.0±8.2	443.9±16.4	n.s.	n.s.	n.s.
-After	479.2±8.1	466.6±11.8	464.8±13.2	454.1±7.6	484.6±14.2	482.4±18.1	n.s.	n.s.	n.s.
-Change	33.9±9.5	58.7±8.9	55.8±9.8	35.6±5.5	43.6±9.1	38.5±5.2	n.s.	n.s.	n.s.
Final total lean (%)	71.0±2.2^c^	79.6±1.7^ab^	71.7±2.2^c^	81.6±1.6^a^	75.5±2.0^bc^	81.7±1.3^a^	*P* < 0.001	n.s.	n.s.
Food intake (g)	615.0±23.1^a^	447.0±14.0^c^	571.6±16.1^ab^	414.3±16.6^c^	531.8±16.0^b^	461.0±19.1^c^	*P* < 0.001	*P* = 0.075	*P* < 0.05
(MJ)	11.67±0.44^a^	8.89±0.28^c^	10.80±0.30^ab^	8.10±0.32^c^	10.11±0.30^b^	9.10±0.38^c^	*P* < 0.001	*P* = 0.05	*P* < 0.05

^1^Body weight and body composition before and after 28-day dietary intervention and cumulative food intake by *ad libitum*-fed DIO rats given high fat diet (HF), HF with added pectin (HF+P), HF with high casein protein (HFHC), HFHC with added pectin (HFHC+P), HF with high pea protein (HFHP), or HFHP with added pectin (HFHP+P). Data are means ± SEM with *n* = 8/group. Two-way ANOVA revealed effects of pectin (+P), protein (HC and HP) and their interaction; n.s., non-significant. Within rows, means with different superscript letters are significantly different.

Daily food intake was in decreasing order of magnitude: HF > HFHC > HFHP > HFHP+P = HF+P > HFHC+P (P<0.001, Fig 1B), with a significant effect of time (*P* < 0.001) but no diet x time interaction beyond the first 6 days of diet acclimatisation. Cumulative food intake was significantly reduced overall by pectin (g and MJ, *P* < 0.001) but not by high protein (g, *P* = 0.075; MJ, *P* = 0.05), with significant pectin x protein interaction (g and MJ, *P* < 0.05) since the effect of pectin was greater with HFHC than HFHP diet (Table 2). Cumulative intake was greatest in the control HF group, no different in HFHC, and lower in the three +P groups (*P* < 0.001); the intermediate value for HFHP was lower than HF (*P* < 0.01) but higher than the +P groups (*P* < 0.001–0.05) (Table 2).

Strong positive correlations were observed between cumulative food intake and weight gain and fat mass change but not lean mass change (Table 3).

**Table 3:** Correlations.

	Intake^1^	BWG	Fat gain	Lean gain	PYY	GLP-1	S.I.	Caecum	Acetate	Propionate	Butyrate
^2^BWG	0.853***										
^3^Fat gain	0.787***	0.917***									
^4^Lean gain	0.010	0.184	0.221								
^5^PYY	-0.538***	-0.597***	-0.555***	-0.021							
^6^GLP-1	-0.500***	-0.471**	-0.447**	0.121	0.440**						
^7^S.I.	-0.125	-0.228	-0.295*	0.120	0.602***	0.368*					
^8^Caecum	-0.702***	-0.731***	-0.782***	-0.012	0.588***	0.646***	0.492***				
^9^Acetate	-0.401**	-0.338*	-0.464***	0.001	0.162	0.330*	0.214	0.483***			
^10^Propionate	-0.415**	-0.360*	-0.432**	-0.062	0.199	0.343*	-0.082	0.449***	0.692***		
^11^Butyrate	0.277	0.307*	0.190	0.064	-0.464***	-0.205	-0.331*	-0.343*	0.280	0.304*	
^12^Succinate	-0.585***	-0.641**	-0.522***	0.148	0.622***	0.485**	0.284	0.585***	-0.026	0.006	-0.508***

Correlations between body weight and composition changes, satiety hormone concentrations, small intestine and caecum weights, and caecal concentrations of SCFAs in DIO rats. ^1^Cumulative food intake ^2^body weight gain, ^3^fat mass gain and ^4^lean mass gain (all g) during the 4-week dietary intervention. Plasma concentrations of ^5^PYY and ^6^total GLP-1 (pg/ml), weight of ^7^S.I. (small intestine) and ^8^full caecum (g), and ^9-12^concentrations in caecal contents (mM) of acetate, propionate, butyrate and succinate after 4-week dietary intervention. Values are Pearson correlation coefficients (*r*)

* *P* < 0.05

**P < 0.01

***P < 0.001.

### Plasma satiety and metabolic parameters

**Fig 2:**
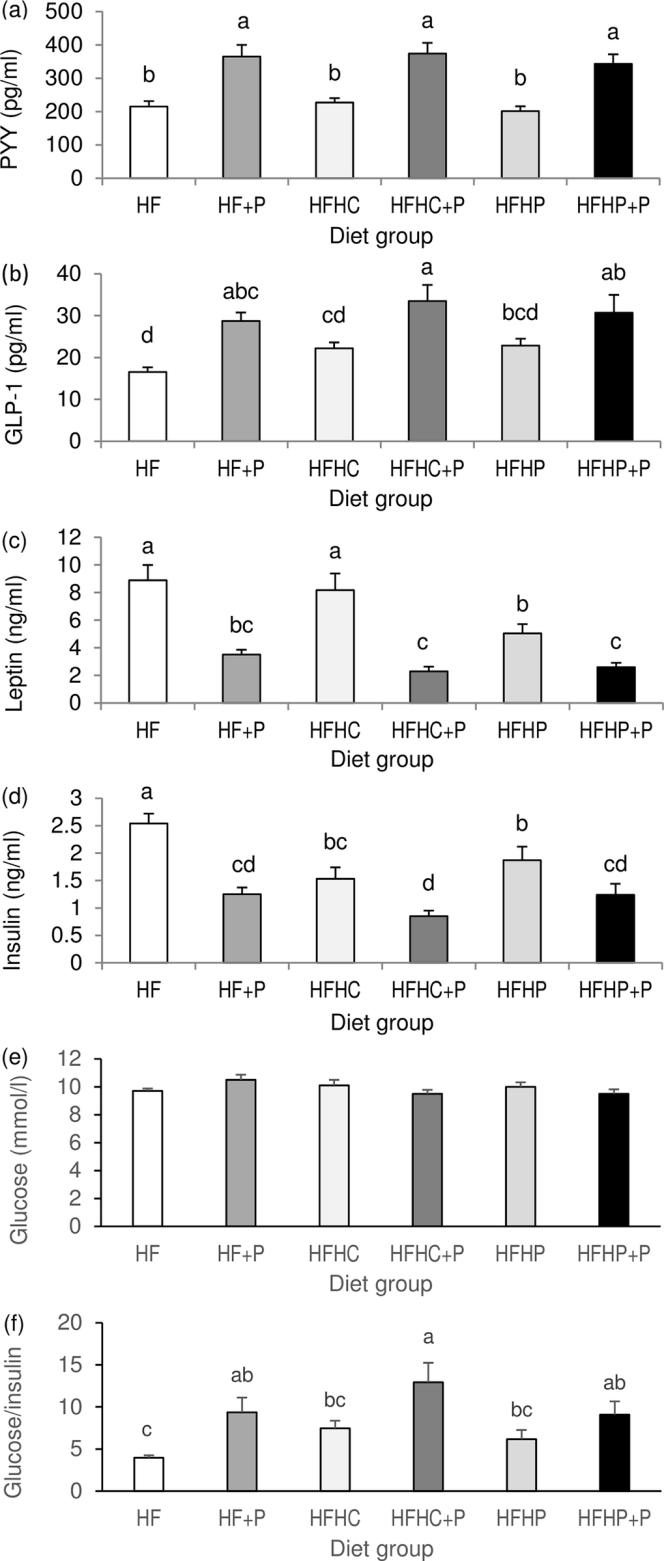
Plasma hormones and glucose. Final plasma concentrations of (a) PYY, (b) total GLP-1, (c) leptin, (d) insulin, (e) glucose, and (f) glucose/insulin ratio in DIO rats fed high fat diet (HF), high fat diet with pectin (HF+P), HF with high casein protein (HFHC), HFHC with pectin (HFHC+P), HF with high pea protein (HFHP), HFHP with pectin (HFHP+P) for 4 weeks (*n* = 8 per group). Statistical analysis by two-way ANOVA with pectin, protein and their interaction as factors, followed by Fisher pairwise comparisons. Within figures, values labelled with different letters are significantly different (*P* < 0.001).

Plasma concentrations of PYY and total GLP-1 were increased by supplementary pectin (both *P* < 0.001) but not by high protein, with no pectin x protein interaction (Fig 2A and 2B). Plasma leptin was decreased by supplementary pectin (*P* < 0.001) and by high pea protein (*P* < 0.05) but not by high casein protein and with no pectin x protein interaction (Fig 2C). Plasma insulin was decreased by pectin (*P* < 0.001) and by high protein (*P* = 0.001) with no pectin x protein interaction (Fig 2D). Plasma glucose was not significantly different between diet groups but the glucose/insulin ratio was increased by pectin (*P* < 0.001) and not by high protein, with no pectin x protein interaction (Fig 2E and 2F).

Strong negative correlations existed between plasma PYY and cumulative food intake and between total GLP-1 and intake (Table 3). Plasma leptin and insulin correlated positively with total body fat mass (*r* = 0.914 and *r* = 0.631, respectively, both *P* < 0.001) and with cumulative intake (*r* = 0.810 and *r* = 0.665, respectively, both *P* < 0.001). Plasma total GLP-1 showed strong negative correlation with plasma insulin (*r* = -0.527, *P* < 0.001) and positive correlation with the glucose/insulin ratio (*r* = 0.455, *P* = 0.002).

### Gut weights

Whole gut weights were not significantly different between the groups, but diet had a significant effect on the weights of the different gut regions. Stomach weight (full) was decreased by supplementary pectin (*P* < 0.01) but not by high protein, with no pectin x protein interaction (Table 4). Small intestine weight and length were increased by pectin (both *P* < 0.001) but not by high protein, with no pectin x protein interaction (Table 4). Weights of full caecum and of caecum contents were increased by pectin and by high pea protein (all *P* < 0.001), with no pectin x protein interaction. Full caecum and contents weights in the HFHP group were higher than in HF and HFHC groups, but lower than in all +P groups, and were greatest in HFHP+P compared with the other +P groups (*P* < 0.001; Table 4). Colon weight was not significantly affected by the dietary treatments.

**Table 4:** Gut morphology^1^.

	Diet						Significance of effects		
	HF	HF+P	HFHC	HFHC+P	HFHP	HFHP+P	Pectin	Protein	Pectin x protein
Whole gut weight (g)	41.0±1.7	40.3±1.2	40.4±2.7	39.0±1.3	38.3±2.3	41.6±2.2	n.s.	n.s.	n.s.
Stomach weight (g)	5.4±0.3^a^	4.4±0.4^ab^	5.5±0.7^a^	3.9±0.5^b^	4.6±0.3^ab^	3.9±0.3^b^	*P* < 0.01	n.s.	n.s.
Small intestine weight (g)	10.8±0.4^c^	12.8±0.6^ab^	11.4±0.7^bc^	12.5±0.6^ab^	10.7±0.6^c^	13.4±0.5^a^	*P* < 0.001	n.s.	n.s.
Small intestine length (mm)	1132±19^cd^	1199±11^bc^	1107±24^d^	1228±24^ab^	1148±14^cd^	1278±38^a^	*P* < 0.001	n.s.	n.s.
Caecum weight -full (g)	3.4±0.3^d^	8.3±0.2^b^	3.1±0.4^d^	8.4±0.6^b^	5.1±0.2^c^	10.0±0.7^a^	*P* < 0.001	*P* < 0.001	n.s.
-empty (g)	1.8±0.1^d^	2.9±0.1^b^	1.6±0.1^d^	3.3±0.2^ab^	2.2±0.1^c^	3.4±0.2^a^	*P* < 0.001	*P* < 0.05	n.s.
-contents (g)	1.7±0.3^d^	5.4±0.2^b^	1.5±0.3^d^	5.2±0.4^b^	2.9±0.1^c^	6.7±0.5^a^	*P* < 0.001	*P* < 0.001	n.s.
Caecum length (mm)	43±2^b^	61±1^a^	42±1^b^	59±2^a^	45±2^b^	59±3^a^	*P* < 0.001	n.s.	n.s.
Colon weight (g)	4.7±0.4	4.1±0.5	4.6±0.6	4.5±0.2	3.6±0.2	4.0±0.3	n.s.	n.s.	n.s.
Colon length (mm)	174±7	178±10	186±7	175±7	161±8	166±4	n.s.	n.s.	n.s.

^1^Final gut regional weights and lengths in diet-induced obese rats fed high fat diet (HF), HF with added pectin (HF+P), HF with high casein protein (HFHC), HFHC with added pectin (HFHC+P), HF with high pea protein (HFHP), or HFHP with added pectin (HFHP+P). Data are means ± SEM with *n* = 8/group. Two-way ANOVA revealed effects of pectin (+P), protein (HC and HP) and their interaction; n.s., non-significant. Within rows, means with different superscript letters are significantly different.

Overall, stomach weight correlated positively with cumulative food intake: (g), *r* = 0.437, *P* < 0.01; (MJ), *r* = 0.426, *P* < 0.01. Small intestine weight correlated strongly with plasma PYY and less strongly though significantly with total GLP-1 (Table 3).

### Caecal SCFAs

**Fig 3:**
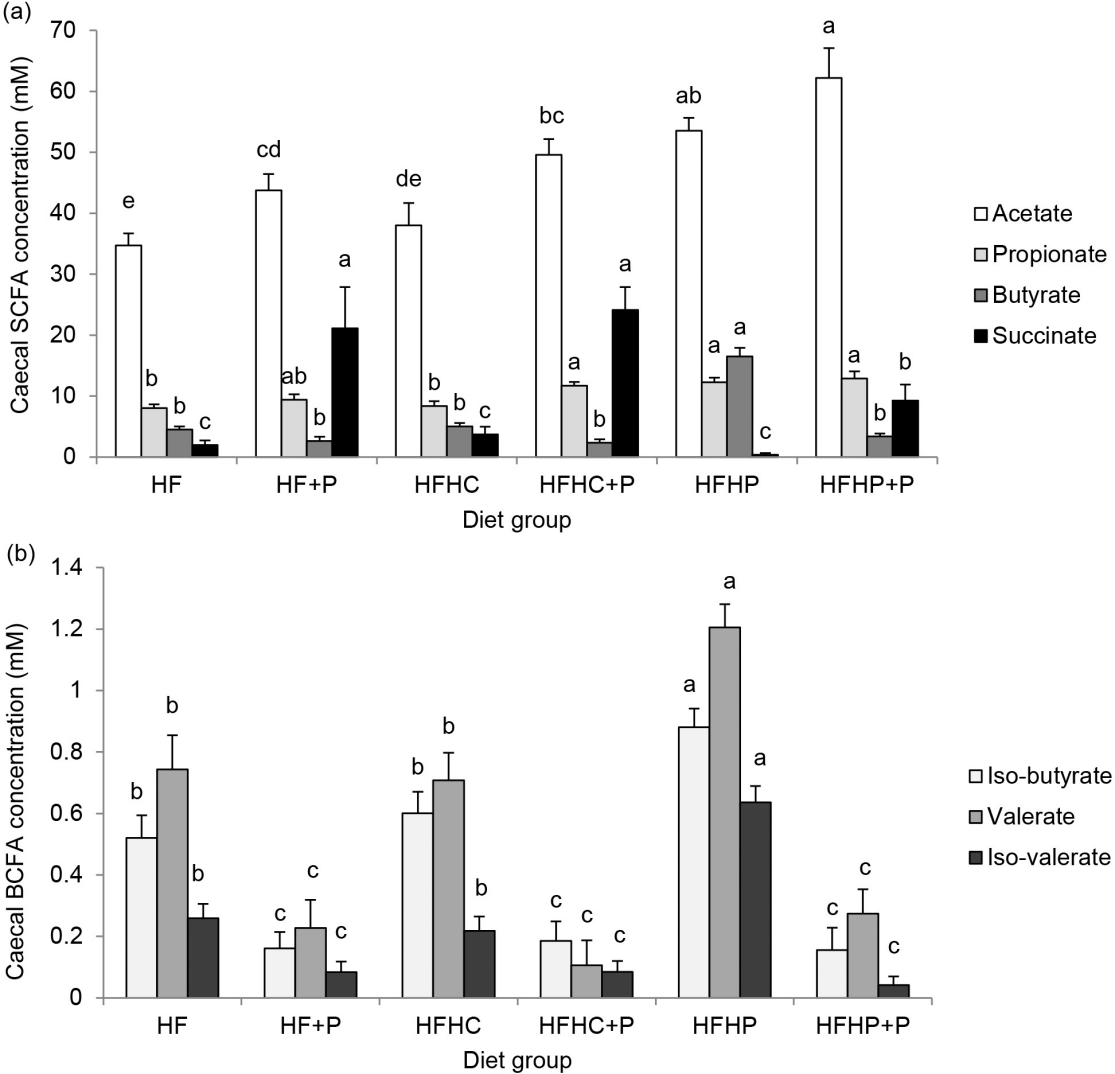
**(a) SCFAs and (b) BCFAs.** Concentrations (mM) of (a) acetate, propionate, butyrate and succinate and (b) iso-butyrate, valerate and iso-valerate in caecal contents of DIO rats fed high fat diet (HF), high fat diet with pectin (HF+P), HF with high casein protein (HFHC), HFHC with pectin (HFHC+P), HF with high pea protein (HFHP), HFHP with pectin (HFHP+P) for 4 weeks (*n* = 8 per group). Statistical analysis by two-way ANOVA with pectin, protein and their interaction as factors, followed by Fisher pairwise comparisons. Within figures, values for a given fatty acid labelled with different letters are significantly different (*P* < 0.001).

SCFA concentrations in caecum contents were increased in the three +P groups and in the HFHP group but not different in the HFHC group compared with HF (Fig 3A). Acetate and propionate concentrations were increased by pectin (*P* < 0.001 and *P* < 0.01, respectively) and by high pea protein (both *P* < 0.001), with no pectin x protein interaction, and no effect of high casein protein. Butyrate was increased by high pea protein (*P* < 0.001), not changed by high casein protein but decreased by pectin (*P* < 0.001), with significant pectin x protein interaction (*P* < 0.001; Fig 3A). Succinate concentrations were specifically increased by pectin supplementation (*P* < 0.001), with no overall effect of protein or any pectin x protein interaction detected, but were lower in HFHP+P than in HF+P and HFHC+P groups (*P* < 0.05; Fig 3A). Concentrations of the BCFAs iso-butyrate, valerate and iso-valerate were decreased by pectin supplementation (all *P* < 0.001) and increased by high pea protein (*P* < 0.05, *P* < 0.01 and *P* < 0.001, respectively), with associated pectin x protein interaction (*P* < 0.05, *P* = 0.05, *P* < 0.001, respectively), and HFHC group values were no different from HF (Fig 3B). Significant negative correlations were detected between food intake and caecal concentrations of acetate, propionate and succinate but not butyrate (Table 3). Acetate, propionate and succinate concentrations correlated positively with caecum weight, but butyrate showed a weak negative correlation. Plasma PYY correlated positively with succinate concentrations, but not acetate or propionate, and negatively with butyrate, while plasma total GLP-1 correlated positively with acetate, propionate and succinate concentrations (Table 3).

## Discussion

The soluble fermentable fibre (pectin) appeared more effective than protein sourced from either animal (casein) or plant (pea) for increasing satiety and decreasing caloric intake and adiposity in DIO rats while still on high fat diet, and also appeared to produce a healthier hindgut environment. To provide some translational perspective, the 10% w/w pectin inclusion rate meant that the rats consumed an estimated 2 g fibre daily in a total energy intake of 380 kJ, which is equivalent to 55 g/d for a man eating on average 10,500 kJ/d, in other words 1.8-fold more than the recommended fibre intake of 30 g/d [29, 30]. The 40% energy protein inclusion rate herein was within the range used experimentally for weight loss in humans [10, 11]. Although the dietary fibre inclusion rate was high, we have previously demonstrated that the satiety and adiposity responses are dose-dependent in rats at lower inclusion rates between 3 and 10% w/w [31]. The implication is that chronic increases in soluble fermentable fibre intake even at lower rates than that used herein may be a better approach than high protein diets for weight loss in obesity, and this is worthy of further investigation in humans.

Both pectin and high pea protein diets decreased body weight gain in the present rats, but pectin had the greater effect associated specifically with significant body fat loss whereas the high pea protein diet only arrested further fat accretion. Lean tissue gain was unaffected so that body composition was markedly different between the diet groups at the end of 4 weeks, with total body fat percentage lower and lean tissue percentage higher in rats fed pectin but not in those fed solely high protein diets. In this study, highly significant correlations indicated that the decreases in weight gain and changes in fat mass were largely attributable to the reduction in voluntary food intake, indicative of a general increase in satiety. In support, background circulating concentrations of the gut-derived satiety hormones PYY and total GLP-1 correlated negatively with food intake. Interestingly, PYY was increased by the increased dietary fibre pectin but not by the increased dietary protein from either source, in line with their respective effects on food intake. Although the lack of effect of high protein diet on PYY secretion contrasts with some published human and rat data, this may be attributable to important differences in study design. Batterham et al [9] studied growing lean mice postprandially and Stengel et al [12] measured increased PYY in DIO rats on high protein (52% energy) diet during a 2-h feeding period in the dark phase, whereas we measured background PYY concentrations during a normal light phase when rats are not feeding [26]. Furthermore, most of the human data report an increase in the acute postprandial PYY response after a high protein meal, without assessing the chronic background PYY levels [32]. The ability of dietary fibre, but not protein, to increase general satiety may be because the former chronically increases background PYY whereas the latter has a more acute effect on PYY. Importantly, in our rat model, PYY concentrations in fibre-fed rats are consistently elevated in both the morning and afternoon of a normal non-feeding light phase (Adam, Thomson and Ross, unpublished).

PYY is secreted in response to the ingestion of food by enteroendocrine L cells that are located, progressively increasing in density, along the distal small intestine and large intestine [33]. However, PYY is initially released within 15 minutes of food intake, which clearly precedes the arrival of ingested nutrients in the distal gut and is therefore thought to be under neural control [34]. This is then followed by further release (about 2 hours after a protein-rich meal, [35]) in response to ingested macronutrients sensed by the L cells [32, 33]. Postprandial PYY stimulation is influenced by the macronutrient content of the meal, in decreasing order of potency protein > fat > digestible carbohydrate, whereas postprandial PYY data are equivocal in a limited number of studies of indigestible carbohydrate (fibre); however, importantly, fasting PYY levels are elevated after chronically increased fibre intakes [32]. The second phase of postprandial PYY release is driven by mechanisms including the activation of specific nutrient-sensing receptors on the L cells [33]. These include receptors for peptones, responding to partially digested protein, but also SCFA receptors responding to the products of hindgut fermentation. Given the more protracted nature of dietary fermentation processes compared with the relatively short term post prandial dietary digestion processes, it follows that fermentable dietary constituents would provide more persistent L cell stimulation, hence increased background inter-meal PYY levels and a general increase in satiety. It is tempting to speculate that to be effective in increasing general satiety and weight loss, an increased amount of hindgut fermentation requires to be maintained by appropriate daily dietary fibre intake. In support, fasting (background) concentrations of PYY are increased in humans taking daily dietary supplements of functional fermentable fibre for several weeks [36–38].

The foregoing discussion has focussed on PYY because it is unequivocally a satiety-signalling hormone and is thought to be the causal mediator in protein-induced satiety [9]. Whilst PYY may indeed underlie short term postprandial protein-induced satiety, the present data indicate that longer term increases in circulating PYY are better sustained by increased dietary fibre intake. By contrast, the physiological status of satiety signalling by GLP-1 is not established, despite its co-secretion by L-cells [39]. Here the background total GLP-1 concentrations were significantly increased along with PYY by the dietary fibre pectin, and may also have contributed to the increased satiety, while the high protein diets led to smaller non-significant increases in plasma total GLP-1. Both the high fibre and high protein diets led to decreased insulinaemia. GLP-1’s highly significant negative correlation with plasma insulin and positive correlation with the glucose to insulin ratio herein were consistent with GLP-1’s established role in glycaemic control [39]. Furthermore, the glucose to insulin ratio was increased (1.8-fold) by dietary pectin, but not by high protein, consistent with improved insulin sensitivity and demonstrating a potential benefit of pectin-enriched diets for the obese. Further beneficial effects on lipidaemia in our DIO rat model have been reported previously [1].

Propionate has been shown in rat and human large intestine to directly stimulate L cell PYY and GLP-1 release, probably via the receptors FFAR2 and 3 [33]. Here, measurements of caecal SCFAs at a single time point although useful could not reveal their levels of turnover which may be of greater relevance. Plasma PYY concentrations did not correlate with the elevated caecal propionate concentrations but did correlate with caecal succinate, which is a precursor in propionate formation by hindgut *Bacteroides* species [18] and which dominated on the high pectin diets. Targeted colonic delivery of propionate conjugated to inulin and mixed in the normal diet produced a 2.5-fold increase in colonic propionate and led to decreased food intake, decreased fat accretion and decreased weight gain in humans [41]. While it might be difficult to achieve such propionate levels in the large intestine solely by increased consumption of fermentable dietary fibre, both targeted colonic delivery and increasing dietary propionate have been mooted as effective approaches for weight management [40, 41]. In addition, in view of the association observed herein and previously by our group [42], it is tempting to speculate that succinate may directly stimulate PYY release. As well as stimulating gut hormone release, acetate and propionate themselves may enter the circulation and directly or indirectly stimulate increased anorexigenic signalling in appetite control centres in the brain [20]. Here, in support, caecal acetate, propionate and succinate concentrations correlated negatively with cumulative food intake in the experimental rats. By contrast, increased caecal butyrate dominated on the high pea protein diet herein but this did not correlate with food intake and correlated negatively with plasma PYY. However, associations have been reported in some mouse models specifically between increased hindgut butyrate production and decreased food intake [43, 44]. The differences between studies may be explained in part by differences in relative dietary composition leading to different patterns of hindgut fermentation. Here, the strong overall negative correlation between caecal succinate and butyrate further highlighted the contrasting pectin versus pea protein fermentation patterns and associated effects on food intake.

The disadvantages of increased hindgut fermentation include the purported undesirable effects of some fermentation products. For example, chronically increased SCFA production in the large intestine may result in chronically increased hepatic portal and systemic SCFA concentrations leading to adverse effects in the liver [45] and kidney [46], respectively. The balance of fermentation products is clearly important for intestinal health [47]. It seems that those produced by fermentation of dietary fibre are generally good for colonic health and colonic cancer prevention [18] whereas those produced from protein fermentation are thought to be detrimental [48], leading to speculation as to whether dietary fibre and protein mixtures might improve the colonic environment [17, 48]. Here, increased protein fermentation was only evident on the high protein diet using pea protein, which increased caecal acetate, propionate and butyrate; the addition of fibre to the diet then seemed to override rather than interact with the fermentation conditions by decreasing the caecal butyrate and increasing succinate. However the lower levels of succinate and higher acetate produced on the high fibre diet with high pea protein compared with high fibre alone does suggest that the protein modified the fibre fermentation by providing more available nitrogen for the fibre-fermenting bacteria [21]. Caecal concentrations of BCFA were only increased by the high pea protein diet, and were decreased compared with controls by the addition of dietary fibre, with or without high protein. As major products of protein fermentation in the gut, BCFAs are associated with poor colonic health and tumour promotion [16, 17], and may therefore be undesirable.

The present data do not provide unequivocal evidence for differential satiation by different protein sources [24, 25]. Food intake was suppressed by pea protein but not by casein, but neither protein increased satiety hormone PYY secretion. However increased pea protein, and not increased casein, was associated with decreased body fat accumulation and increased caecal SCFAs and BCFAs. These differences were clearly attributable to differences in protein digestibility and generalisations comparing animal versus plant protein cannot be made from the present data. Casein is more highly digestible than pea protein [49] and there was no evidence for increased hindgut fermentation on the high casein diet whereas undigested pea protein reaching the large intestine did provide substrate for fermentation.

The diets leading to increased fermentation in the present trial had significant effects on gut morphology. Thus both the high fibre and high pea protein diets increased caecum size, and the combination of high fibre and high pea protein had the greatest (additive) effect on caecum size, likely reflecting the greatest mass of fermenting microbiota. The high fibre diets herein also increased small intestine size, which may be attributable to a decreased transit time, since these effects have previously been linked in rats fed non-fermentable algal polysaccharide [50]. Dose-sensitive effects of dietary fibre (pectin) on increasing apparently healthy caecum and small intestine tissue weights have previously been reported in rats [31]. While the greater gut weight itself may not necessarily be desirable, the increased fibre fermentation within the lumen may produce a healthier internal colonic environment, as discussed earlier. Furthermore, the impact of the present diets on the gut microbiota clearly deserves further exploration, given the differences in fermentation products observed and the known link between gut microbiota and body weight [51].

Leptin and insulin decreased according to decreases in relative fat mass, indicative of improved metabolic health, especially in fibre-fed DIO rats. However, there was no evidence for the decreased secretion of these anorexigenic hormones providing any influence on appetite drive. As previously noted in our fibre-fed DIO rat model [1], the coincidence of increased anorexigenic PYY secretion but decreased anorexigenic hormones leptin and insulin indicates there may be a hierarchy of influence. Gut satiety hormone (PYY) signalling may dominate over metabolic hormone feedback to hypothalamic appetite regulatory pathways, or the central leptin and insulin resistance associated with DIO may persist [52, 53].

In summary, our results indicate that pectin supplementation had the most obvious benefits on satiety, body weight, body composition and gut fermentation in obese rats feeding on high fat diet. Incorporation of pea protein into the high fat diet also had some minor benefits, but combining pea protein and pectin did not offer any advantage over pectin alone. In other words, soluble fermentable fibre appears more effective than high protein for decreasing caloric intake and adiposity while on high fat diet, and producing a fermentation environment more likely to promote hindgut health. Altogether the data support the concept that high fibre may be better than high protein for healthy weight loss in obesity.
